# Family Structure and Early Home Leaving: A Mediation Analysis

**DOI:** 10.1007/s10680-017-9461-1

**Published:** 2018-01-24

**Authors:** Lonneke van den Berg, Matthijs Kalmijn, Thomas Leopold

**Affiliations:** 1 Department of Sociology, University of Amsterdam, Nieuwe Achtergracht 166, 1018 WV Amsterdam, The Netherlands; 2 Netherlands Interdisciplinary Demographic Institute (NIDI), The Hague, The Netherlands

**Keywords:** Leaving home, Family structure, Young adulthood, Transition to adulthood

## Abstract

An ample body of research has shown that young adults from non-intact families are more likely to leave the parental home at an early age than young adults from intact families. However, little is known about the mechanisms underlying this relationship. We drew on prospective longitudinal data from the German Socio-Economic Panel Study (SOEP) to examine why young adults from non-intact families are more likely to leave home early. Based on the feathered nest hypothesis, it was expected that young adults from non-intact families are pushed out of the parental home because of a lack in economic, social, and community resources. Moreover, it was expected that young adults from non-intact families are pulled toward independent living at a younger age because they have a partner and are employed earlier in life. We employed discrete-time event history models and used the KHB method to test relative weights of the mediators. The mediators explained 16% (women) and 22% (men) of the effect of living in a stepfamily, and 50% (women) and 37% (men) of the effect of living in a single-mother family. Economic resources were the main mediator for the effect of living in a single-mother family on early home leaving. For women, mother’s life satisfaction and housing conditions significantly explained differences in early home leaving between single-mother and intact families. For men, residential mobility significantly mediated the effect of family structure on early home leaving.

## Introduction

Leaving the parental home to start an independent household is considered a milestone in the transition to adulthood. This milestone is characterized by heterogeneity in timing. Some leave home before age 18, others well above age 30. An important factor explaining heterogeneity in the age at leaving home is family structure. Young adults from “non-intact” families leave home at a younger age than young adults from “intact” families in which both biological parents are present (e.g., Aquilino 1991; Goldscheider and Goldscheider 1998; Raab 2017; Sandberg-Thoma et al. 2015).

Although the effect of family structure on the age of leaving home is well-documented, we know little about how it can be explained. The aim of this research is to analyze mediation of the effect of family structure on early home leaving to assess the relative weight of different mediators. The present study also examines whether there are differences in the mediation of the effect of living in a stepfamily and of living in a single-mother family on early home leaving. Specifically, we test the feathered nest hypothesis, which posits that young adults from non-intact families have less economic, social, and community resources and hence are more likely to be pushed out of the parental home at a younger age (Avery et al. 1992). Moreover, we examine mediation through pull factors toward independent living, because young adults from non-intact families experience union formation (Ivanova et al. 2011) and leave the educational system (Amato 2001) earlier in life.

It is important to study why young adults from non-intact families are more likely to leave home at an early age, because early home leaving as opposed to normative and late home leaving is considered an explanation of some of the harmful effects of living in a non-intact family on children’s outcomes in later life. Previous research has demonstrated that early home leaving is associated with several poor outcomes in adulthood. First, early home leavers have fewer possibilities to draw on resources from their parental home. Early leaving is the most important predictor for living in poverty during young adulthood (Aassve et al. 2007) and increases the chance of having debt problems (Oksanen et al. 2016). Second, early home leaving is associated with early union formation. These unions, in turn, are more likely to end in divorce or separation (Lehrer 2006). Third, previous research found that young adults who leave the parental home at an early age are less close to their parents later in life than young adults who leave home at a normative or late age (Leopold 2012; Tosi and Gähler 2016). Hence, early home leaving might be harmful for intergenerational ties later in life.

We use discrete-time event history models drawing on longitudinal data from the German Socio-Economic Panel Study (SOEP) to examine to what extent push and pull factors explain early home leaving among young adults from non-intact families. Using the method developed by Karlson et al. (2012) to test mediation, we assess not only whether these factors mediate the relationship between family structure and early home leaving, but also their relative weights. We distinguish between two types of non-intact families, single-mother families and stepfamilies. We combine prospective data on family structure, employment, income, and housing conditions of young adults and their mothers with detailed data on social relations from a youth questionnaire answered in the year the young adult turned 17. These rich data allow us to examine a wider range of mediating factors than previous research.

## Theoretical Background and Hypotheses

### Leaving Home in Germany

Young adults in Germany leave home relatively early compared to Eastern and Southern European countries, but slightly later than in Northern European countries and the USA (Mitchell 2007, p. 66). In Germany, the median age at leaving home was 22.4 for men and 20.6 for women born around 1960 (Billari et al. 2001).

Several factors are important to understand how leaving home in Germany compares to other countries. First, Germany is a conservative welfare state, offering public assistance through student loans and housing allowance (Berngruber 2015; Billari et al. 2001). Second, Germany has the largest rental market in Europe, with an important private rental sector (Dol and Haffner 2010). Hence, young adults face relatively few economic constraints on leaving home. In line with this, Mulder et al. (2002) found a weaker effect of employment status on leaving home in West Germany than in the USA. Third, leaving home for college is less common in West Germany than in the USA (Mulder et al. 2002). This could be explained by the smaller size of the country, allowing more young adults in Germany to commute to university.

### Previous Research on Family Structure and Leaving Home

Ample research has documented a link between family structure and early home leaving (Aquilino 1991; Bernhardt et al. 2005; Goldscheider and Goldscheider 1998; Raab 2017; Sandberg-Thoma et al. 2015). Most of these studies have examined the effect of family structure on the average age of leaving home (Bernhardt et al. 2005; Goldscheider and Goldscheider 1998; Sandberg-Thoma et al. 2015), fewer on the odds of early home leaving (Aquilino 1991; Raab 2017). Both lines of research found a medium-sized effect of family structure. In general, these studies suggested that differences in the age of leaving home are most pronounced between young adults from stepfamilies and those from intact families (Aquilino 1991; Bernhardt et al. 2005; Blaauboer and Mulder 2009; Goldscheider and Goldscheider 1998; Iacovou 2010).

To our knowledge, only two previous studies examined mediation of the relationship between family structure and leaving home. Cooney and Mortimer (1999) examined several mediators (family size, family income, parent–child relationship, housework hours, income of the young adult, psychological efficacy, peer involvement) in an early study using the American Youth Development Study. Lois (2014) studied multiple mediators (economic deprivation, social control, instability, stress) of the relationship between family structure and early home leaving using data of the German Family Panel. We add to these previous studies in various ways. First, we add several relevant mediators such as experiencing residential mobility, having a partner, life satisfaction of the mother, enrollment in education by education level, and more refined indicators such as housing conditions for economic well-being. Second, we examine all mediators for step- and single-mother families more systematically and assess the relative weight of each of the mediators. Third, in light of strong gender differences in the timing of leaving home we conduct our analyses separately for men and women.

### Push Factors: Resources in the Parental Home

#### Parental Economic Resources

Young adults from non-intact families are more likely to come from economically deprived parental homes—characterized by poverty and poor housing conditions—than young adults from intact families (Andreß et al. 2006; Dewilde and Stier 2014; Uunk 2004). Economic resources in single-mother families are limited due to women’s lack of experience in the labor market, competing demands for child care, selection into single parenthood/divorce, loss of an earner, and loss of economies of scale. Mothers who repartner are often able to overcome some, but not all of these financial constraints (Sweeney 2010; Uunk 2004).

Parental income “feathers” the parental nest by providing the young adults with material comfort, sustaining their consumption, and supporting them financially. Research has shown that this financial support is not fully transferable; parents are more likely to support co-resident children than children who left home (Angelini and Laferrère 2012). Parents with low incomes have limited possibilities to support their children and might instead experience the economic burden of a co-residing child. Hence, these parents might “push” their children out of the home at an early age by asking them to move out or, less directly, by providing less material comfort that “feathers” the home (Cooney and Mortimer 1999).

The parental home might also be more feathered in terms of housing quality. Good housing conditions increase the quality of life in the parental home and provide more privacy to the child (e.g., own room, own bathroom). In contrast, poor housing conditions may render staying at home undesirable and push the child out of the parental home.

Most previous studies showed that poor housing conditions (Buck and Scott 1993; Mulder et al. 2002) and low income (Blaauboer and Mulder 2009; Buck and Scott 1993; Mulder et al. 2006) indeed increase earlier home leaving. The effect of parental income is dependent on age; high parental income prevents early home leaving, but promotes leaving at later ages (Avery et al. 1992; Iacovou 2010).

Based on the feathered nest hypothesis, we hypothesize the following: *Early home leaving among young adults from non*-*intact families is mediated by economic resources in the parental home (Hypothesis 1a).* As mothers who repartner are able to overcome some of their financial problems (Uunk 2004), we further expect that *parental economic resources mediates more of the effect of single*-*mother families than of stepfamilies (Hypothesis 1b).*

#### Social Resources

Young adults from single-mother and stepfamilies also face disadvantages in terms of social resources in the parental home—characterized by the quality of the relationship to the mother, emotional support, and parental educational support. Research has found that compared to children in intact families, children from non-intact families were less close to their parents (Afifi and Schrodt 2003), received less parental support in school (Ressler et al. 2017), less often regarded their parents as a source of help or support, and felt fewer obligations toward helping their stepparents than their parents (Amato et al. 1995; Kalmijn and Dronkers 2015).

For both single-mother families and stepfamilies this might first be explained by a selection effect. Mothers who have difficulties in maintaining close relationships might be more likely to divorce or never marry, and to have a weaker relationship with their child. Secondly, in single-mother families the mother has to fulfill two roles, she is both the breadwinner and responsible for all parenting tasks. Hence, children in single-mother families receive less time and monitoring (McLanahan and Sandefur 1994). A stepfather might not fully substitute for the original parent, as stepfathers are often less committed to the child (McLanahan and Sandefur 1994). Moreover, in stepfamilies, the presence of a stepfather could disrupt social relations in the parental home and add to stress, as the child has to adjust to the new family situation and to the stepparent (King 2009; McLanahan and Sandefur 1994; Sweeney 2007). Lastly, the life satisfaction of single mothers, but also of repartnered mothers, is lower than the life satisfaction of continuously married mothers (Carr and Springer 2010). Lower life satisfaction among mothers from non-intact families could have deleterious effect on parenting behaviors and result in a reduced level of emotional availability for the child (Kalmijn and Dronkers 2015; Wilson and Durbin 2010).

The feathered nest hypothesis might also apply to non-economic resources that make the parental home more attractive to live in. Company and support of parents are mostly non-transferable resources that would be (partially) lost if the young adult moves out. Conversely, parental homes with high levels of stress might push the child out of the home (Amato and Kane 2011). Previous research has shown the importance of parent–child relationships for leaving home. Young adults who were less close to their parents or had a conflict with their parents were more likely to leave home (Bernhardt et al. 2005; South and Lei 2015) and to have left home at a young age (Cherlin et al. 1995; Cooney and Mortimer 1999). Research based on retrospective data on reasons for early home leaving found that young adults from non-intact families were more likely to have left due to friction than young adults from intact families (Cherlin et al. 1995).

Based on these considerations, we hypothesize the following: *Early home leaving among young adults from non*-*intact families is mediated by social resources in the parental home (Hypothesis 2).* We do not expect a difference between single-mother and stepfamilies in the mediation effect of social resources, as the stepparent might disrupt the mother–child relationship, but might also enhance the mother’s life satisfaction.

#### Community Resources

Young adults from non-intact families might also have fewer community resources than young adults from intact families (McLanahan and Sandefur 1994). In this study, we use an indirect measure of community resources, namely residential mobility. Residential mobility has often been linked to community resources, because moving to a different area could disrupt access to community resources and decrease (local) social capital (Coleman 1988; Hagan et al. 1996). Individuals who live in an area for a longer period have better access to its resources through networks of friends and acquaintances (Goldscheider and DaVanzo 1989; Keene et al. 2013; McLanahan and Sandefur 1994).

Young adults from non-intact families are more likely to have experienced residential mobility in childhood. Non-intact families are more likely to be mobile because family transitions such as union dissolution and remarriage often involve moving (Sweeney 2007). Stepfamilies have the highest residential mobility rate, as both the dissolution of the union and repartnering are associated with moving.

The feathered nest hypothesis could also be applied to community resources. The parental home might be less attractive to stay in for young adults who have experienced residential mobility because they have fewer ties to the neighborhood. Moreover, residential mobility experienced in childhood is predictive of later residential mobility due to socialization and status inheritance (Myers 1999). Hence, young adults who have experienced residential mobility might leave the parental home at an earlier age (Goldscheider and DaVanzo 1989; Hill et al. 1996) and move across shorter distances (Leopold et al. 2012). To our knowledge, residential mobility has not yet been examined in relation to family structure and leaving home. However, the findings of previous research showed that residential mobility (partially) mediated the relationship between family structure and other children’s outcomes such as school attainment (e.g., Astone and Mclanahan 1994; McLanahan and Sandefur 1994). In line with the feathered nest hypothesis, we test the following mediation hypothesis: *Early home leaving among young adults from non*-*intact families is mediated by residential mobility (Hypothesis 3a).* Moreover, as the number of family transitions is higher in stepfamilies than in single-mother families, we expect that *residential mobility mediates more of the effect of stepfamilies than of single*-*mother families (Hypothesis 3b).*

### Pull Factors

Young adults might not only leave home because they are pushed out of the parental home, they might also leave because they are pulled toward independent living. We examine two pull factors that might render independent living more desirable.

#### Main Activity: Education and Employment

Parents from non-intact families might also have fewer economic and social resources—previously discussed as push factors—to invest in their children’s education (e.g., helping with homework, hiring a tutor). As a result, education and employment might differ between young adults from intact and non-intact families, with young adults from non-intact families leaving education at a younger age and having a lower educational attainment (Amato 2001; Francesconi et al. 2010; Bernardi and Boertien 2017; Gähler and Palmtag 2015).

Leaving the educational system is associated with a role transition for young adults (Arnett 2000), pulling them toward independent living. Upon finishing education, young adults who are unemployed might leave home to find employment (Holdsworth and Morgan 2005). Young adults who are employed might also leave home, as independent living becomes more affordable. Previous research has shown earlier home leaving among young adults who were employed compared to those who were still in education (Avery et al. 1992; Iacovou 2010). In our analysis, we test the following mediation hypothesis: *Early home leaving among young adults from non*-*intact families is mediated by earlier leaving the educational system and earlier labor market entry (Hypothesis 4).* We do not expect a difference in the strength of the mediating effect of main activity between single-mother families and stepfamilies, as previous findings suggest no differences in education between non-intact family types (Gähler and Palmtag 2015).

#### Having a Partner

Young adults from non-intact families might also be pulled toward early home leaving through early union formation. Research has shown that young adults from non-intact families date, marry, and cohabit earlier (Amato and Kane 2011; Ivanova et al. 2011; Kalmijn and Dronkers 2015). This applies especially to children in stepfamilies, less to children in single-mother families. Young adults from non-intact families have fewer economic resources and are less often enrolled in higher education, which speeds up the process of family formation (Amato and Kane 2011). Moreover, young adults from non-intact families might adopt non-traditional family views through modeling behavior. Having a partner might not only promote the wish to move out, but also vice versa: Young adults who would like to leave home might see a partnership as a viable route out of the parental home.

Several studies showed that young adults who have a partner are more likely to leave home (Mulder and Hooimeijer 2002; South and Lei 2015). The higher likelihood of having a partner among young adults from non-intact families and the pull effect of having a partner motivate our final mediation hypothesis: *Early home leaving among young adults from non*-*intact families is mediated by earlier union formation (Hypothesis 5a).* As union formation is early especially among young adults in stepfamilies, we expect that *having a partner mediates more of the effect of stepfamilies than of single*-*mother families (Hypothesis 5b).*

## Method

### Data

Our analyses were based on data from 15 waves (2001–2015; SOEP-long v32-1, release 2017) of the German Socio-Economic Panel Study (SOEP), see https://www.diw.de/en/soep. The SOEP is an annual household and person survey that contains both retrospective and prospective information (Wagner et al. 2007). Since 2000, children of SOEP participants answer a youth biography questionnaire in the year in which they turn 17. The youth biography questionnaire contains a wide range of questions on topics relevant for youth, such as their schooling, activities, and social life (since 2001). Additionally, we used information provided by the young adults as regular respondents of the SOEP in subsequent years, information provided by the mother in preceding and subsequent years, and information on the economic conditions of the household through the household questionnaires answered by the head of the household.

### Sample

In total, 6146 young adults have participated in the youth biography questionnaire since 2001. We proceeded in five steps to select an analytical sample. First, we dropped individuals (*n* = 1181) who were observed only once and for whom we did not know whether they moved out after they stopped participation in the SOEP. Second, we dropped individuals (*n* = 208) for whom we did not have information about their mother or who did not live with their mother at first observation. This group consisted of young adults whose resident parent did not participate (*n* = 9), who lived only with the father (*n* = 166) or who were not living with the parents at first observation (*n* = 33). Third, we dropped individuals (*n* = 77) who grew up in non-intact families due to widowhood. Young adults from non-intact families due to widowhood and from families headed by the father were dropped from the sample because they might differ in the dependent and/or independent variables from the other types of non-intact families. The groups were too small to consider as separate categories. Additional analyses showed that these young adults were also more likely to leave home early than those in intact families. However, their resources differed from the other non-intact families; their families had more economic resources than mother-headed families through single parenthood or divorce. Fourth, we dropped individuals outside our age bounds, who were older than 21 at the second observation (*n* = 8). Finally, we dropped individuals (*n* = 126) who had missing information on one of our key variables and for whom we could not use a lagged version of that key variable. Our final sample consisted of 4546 individuals.

### Dependent Variable

Our dependent variable was *early home leaving*. We defined early home leaving as leaving the parental home before age 20 for women and before age 22 for men. We used a different cutoff age for women and men because of the strong gender differences in age at leaving home. There were two reasons for focusing on early home leaving rather than the entire process of home leaving. First, especially early home leaving entails deleterious effects on later-life outcomes, such as poverty and divorce. Second, family structure mainly affects early home leaving. Survivor functions estimated separately by type of family structure showed that differences in the timing of home leaving between intact and non-intact families decreased at higher ages. We did robustness checks with cutoff ages between 20 and 23 for both women and men. Our findings show no notable differences in the main findings and in the mediation analyses between the analyses with these different cutoff ages. It should be noted that 52 of the young adults who left home returned home in subsequent years. We did not exclude these cases from our analyses because we were interested in why young adults leave home (initially) rather than whether they stayed out of the parental home.

We defined a young adult’s move out if his or her household identification number (which is shared by all members living in one household) changed across two subsequent waves and the young adult was no longer identified as child of the head of the household. Young adults might be more likely to drop out of the SOEP when they leave home. To prevent restricting home leaving to a possibly selective group of individuals, we used household data provided by the mother in these cases. We added a wave for these individuals for the year they first dropped out, based on the household data of the mother for the leaving home variable and the young adults’ values on the independent variables in the last wave they participated for the lagged predictors. Individuals who dropped out of the SOEP after having participated in the youth questionnaire were coded as having left home early if their mother continued participating in the SOEP and the number of household members aged 15 and older declined in the wave of drop out due to another cause than the death of a child. Young adults who dropped out of the SOEP were coded as not moved out of the parental home if based on the household data provided by the mother the number of household members aged 15 and older did not decline in the wave of drop out. A robustness check with an exit survey among 54 young adults who stopped participation in the SOEP showed that all young adults who filled in the exit survey and whom we had coded as having left home had indeed left home.

### Independent Variables

Most independent variables were time-changing and lagged by one survey year. Two of the social resources measures (quality of the mother–child relationship and parental involvement in school) and the measure for residential mobility were based on the youth questionnaire and time constant.

#### Family Structure

Our measure of family structure was based on the marital status of the mother, measured with retrospective questions on her marital history at panel entry and prospective information on marital status and the resident partner’s identification number. Moreover, we used a question from the youth questionnaire on the number of years the young adult lived together with both biological parents in the first 15 years of his or her life. We defined the mother’s family structure as intact if the mother was married or living together with a partner when the child was born and was still married or living together with the same partner at the current wave. If the mother started participation in the SOEP after the child was born and was not married when the child was born, the family structure was coded as intact if the mother lived together with a partner at first observation and the child reported to have lived together with both biological parents throughout all of his or her first 15 years of life. Individuals were defined as living in a stepfamily if the mother was married or living with a partner who was not identified as the parent of the child (based on data provided by the mother on her resident partner around birth and early childhood). Individuals were coded as living in a single-mother family if the mother was not married and not living together with a partner. Note that widows were dropped, so both single-mother families and stepfamilies were not intact for other reasons than death of a parent.

#### Parental Economic Resources

##### Household Income

We measured annual post-government income of the parental household as the sum of total family income from labor earnings, asset flows, retirement income, private transfers (including alimony and child support payments), public transfers (including housing allowances, child benefits, subsistence assistance, maternity benefits, unemployment benefits), and social security pensions minus family taxes. We have used the square root scale to adjust income for household composition; we divided the income measure by the square root of the household size. Next, we recoded this variable into four categories indicating the percentage of the median income (less than 60%, 60–100%, 100–150% (reference), and more than 150%) in the respective survey year calculated for the full sample of the SOEP. This categorical specification included a measure for poverty (the bottom category corresponding to the European Commission definition of poverty) and accounted for possible nonlinear effects of income.

##### Housing Conditions

This variable was based on the following question in the household questionnaire: “How would you describe the condition of the building you live in?” This variable was coded as a dummy variable with “good condition” as the reference category and “in need of renovation/state of collapse” coded as one.

##### Homeownership

Homeownership could indicate housing conditions and attachment to the house, as homeowners might be more willing to put effort into housing maintenance than tenants. A dummy variable was coded as one if the head of the household owned the home.

#### Social Resources

##### Quality of the Mother–Child Relationship

The quality of the relationship with the mother was measured by eleven questions covering different aspects. Examples of these questions are: “How important is your mother in your life?” “How often do you talk to your mother about things that worry you?” “How often do you argue or fight with your mother?” Young adults answered on a 4-point Likert scale (*very important* to *unimportant*) and a 5-point Likert scale *(very often* to *never).* We standardized the items over the full sample of the youth questionnaire. This enabled us to determine the relative position of the young adult compared to others rather than the absolute score on each item, and to construct a scale even in the presence of missing values. The scale was the standardized average score of the valid standardized items. The reliability of the scale was *α* = .82. A higher score on this scale indicates a better relationship with the mother.

##### Parental Involvement in School

Parental involvement in school was measured by two questions in the youth questionnaire asking whether the parent(s) showed interest in school and helped with homework. The questions did not specify which parent figures were included. So, the child’s answer might reflect the parental investment of both residential and non-residential parent(s). Parental school involvement was dummy-coded as “not involved” if the parent(s) did not show interest in school and did not help with homework.

##### Mother’s Life Satisfaction

The life satisfaction of the mother was measured annually with the following question: “How satisfied are you with your life, all things considered?” Individuals rated their life satisfaction on an 11-point scale ranging from 0 (*completely dissatisfied*) to 10 (*completely satisfied*). The scale was centered at the sample mean.

#### Community Resources

##### Residential Mobility

As a proxy for community resources and the presence and strength of neighborhood ties, we used an indicator for residential mobility. Young adults were asked whether they lived in a large city, a small city, a large village, or a small village for the largest share of the first 15 years of their life. Next, they were asked if they were still living in that area. A dummy variable was coded one if the young adult was residential mobile—if he or she had moved away from the place of childhood residence.

#### Pull Factors

##### Main Activity

Young adults who were employed (part-time or full-time) and were no longer in education were coded as the reference category. Young adults who were still in education were coded according to the level of education in which they were currently enrolled (general education, vocational education, higher education). A fifth category was composed of young adults who did military service or a voluntary social year. A sixth category consisted of young adults who were not in education and not employed.

There are different pathways out of the parental home. Young adults could leave for union formation, to start education, or to start employment. Unfortunately, it was not possible to account for these pathways. However, this measure was used to account for some of the pull factors toward independent living—the possible pathways out of the parental home. Moreover, we did a robustness check in which we excluded young adults in higher education in order to assess the effect of family structure on early home leaving for other reasons than education. This check supported our main findings.

##### Having a Partner

This measure was based on the following question: “Are you in a serious/permanent relationship?” Individuals who answered yes were coded as having a partner, and those who answered no were in the reference category.

#### Control Variables

##### Location

Age of leaving home and family structure differ between East (former German Democratic Republic) and West Germany (former Federal Republic of Germany). Young adults from East Germany are more likely to leave early than young adults in West Germany (Raab 2017). The divorce rate in East Germany is also higher than in West Germany (Juang et al. 1999). To control for these effects, we used a variable to indicate whether the mother was living in East or West Germany in 1989 (before reunification).

##### Number of Children in the Household

The number of household members younger than 18—outside the young adult him/herself—was included as a control variable to account for sharing housing and income with household members other than the parents.

##### Year of First Observation

The year of first observation was included as a linear control variable to account for possible period effects. Robustness checks with dummies for calendar year yielded similar results.

### Models

We estimated discrete-time event history models for the process of home leaving. We truncated the sample after age 20 for women and age 22 for men because the focus was on early leaving. We first estimated the main effect of family structure on the probability of leaving home and subsequently added the push and pull factors. Mediation effects cannot be examined by comparing log-odds or odds ratios of different models, because rescaling occurs when the model changes. Karlson et al. (2012) introduced a method that allowed us to examine mediation effects in logistic probability models. We used this method to estimate mediation effects and to assess the relative weights of the mediators.

## Results

### Descriptive Analysis

**Fig. 1:**
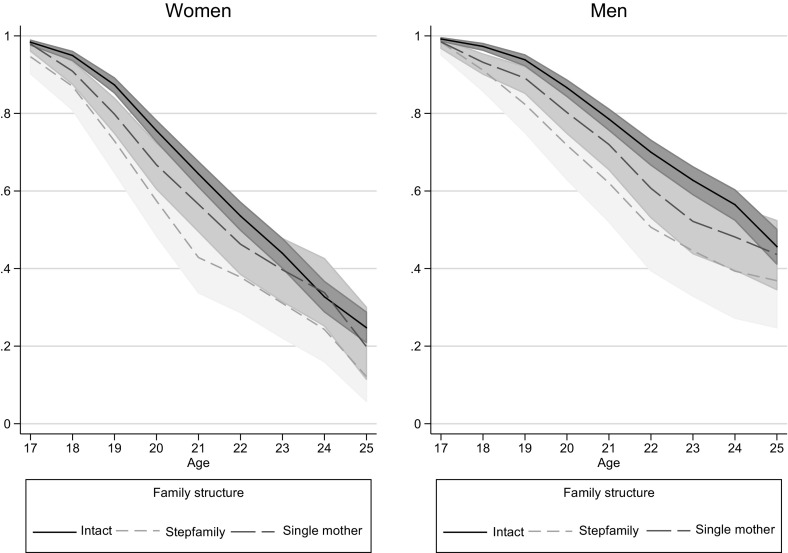
Survival curve for leaving home by family structure. *Source*: German Socio-Economic Panel 2001–2015, own calculations

Figure 1 gives the survivor curves for the process of leaving home for women (left-hand panel) and men (right-hand panel). The figure shows that both women and men from non-intact families left home earlier than those from intact families. This difference was the largest for young adults from stepfamilies. The gap in early home leaving between non-intact and intact families increased until age 21 for women and until age 23 for men.

Table 1 shows descriptive statistics for our analytical sample. Statistics on the independent variables pertain to the initial observation (i.e., upon completion of the youth questionnaire), statistics on leaving home pertain to the first wave after the youth questionnaire. In line with previous research, we found that young adults from non-intact families, especially from single-mother families, had fewer parental economic resources. About 40% of young adults from single-mother families lived below the poverty line—compared to 7% of young adults from intact families. Young adults from single-mother families were also more likely to live in poor housing conditions than young adults from intact families (45% compared to 29%) and were less likely to live in an owner-occupied housing unit (23% compared to 70%).

**Table 1:** Descriptive statistics on the total sample of young adults in Germany by family structure. Leaving home is measured at age 18; all other measures are measured at first observation (age 17). *Source*: German Socio-Economic Panel 2001–2015, own calculations

	Women					
	Intact		Stepfamily		Single mother	
	Mean (%)	SD	Mean (%)	SD	Mean (%)	SD
Left home	2.53		8.42		4.07	
Household income (% of median income)						
< 60%	7.72		12.63		42.31	
60–100%	36.50		38.95		40.27	
100–150%	32.00		27.89		14.48	
> 150%	23.78		20.53		2.94	
Homeowner	69.90		43.20		22.90	
Poor housing conditions	30.00		34.70		45.90	
Quality of mother–child relationship	0.08	0.91	− 0.01	0.98	0.04	1.02
Parent(s) not involved in school	11.40		11.60		16.30	
Life satisfaction parent	7.15	1.68	6.88	1.82	6.41	2.07
Experienced residential mobility	7.04		18.90		13.80	
Main activity						
Employed	0.93		1.58		1.81	
General education	80.91		80.00		77.15	
Vocational education	15.69		16.32		17.65	
Higher education	0.31		1.05		0.23	
Military/social year	0.12		1.05		3.17	
Not employed, not in education	2.04		0.00		0.00	
Having a partner	60.20		70.50		62.90	
Number of children in household	1.06	1.14	0.96	1.01	0.77	0.88
Part of Germany						
East	19.39		37.37		24.43	
West	71.03		58.95		70.59	
Abroad	9.57		3.68		4.98	
First survey year	2008	4.25	2007	4.15	2010	3.89
*N*	1618		190		442	

	Men					
	Intact		Stepfamily		Single mother	
	Mean (%)	SD	Mean (%)	SD	Mean (%)	SD
Left home	1.58		4.66		4.40	
Household income (% of median income)						
< 60%	6.25		11.40		38.02	
60–100%	37.60		40.93		45.93	
100–150%	34.87		29.02		13.19	
> 150%	21.29		18.65		2.86	
Homeowner	70.50		39.90		24.20	
Poor housing conditions	28.00		40.40		45.50	
Quality of mother–child relationship	− 0.04	0.85	0.00	0.91	− 0.03	0.93
Parent(s) not involved in school	9.28		14.00		14.30	
Life satisfaction parent	7.13	1.71	6.90	1.81	6.60	1.95
Experienced residential mobility	5.70		17.60		11.90	
Main activity						
Employed	2.06		0.00		3.74	
General education	77.38		71.50		75.82	
Vocational education	17.89		26.42		17.14	
Higher education	0.18		0.00		0.22	
Military/social year	0.12		2.07		0.00	
Not employed, not in education	2.37		0.00		3.08	
Having a partner	47.60		56.00		51.40	
Number of children in household	1.00	1.05	0.93	1.07	0.72	1.02
Part of Germany						
East	20.86		29.02		22.42	
West	71.26		65.80		74.07	
Abroad	7.88		5.18		3.52	
First survey year	2008	4.12	2009	4.11	2009	4.09
*N*	1648		193		455	

Young adults from non-intact families differed less from young adults living in intact families in terms of social resources. In contrast to our expectations and previous research, the quality of the mother–child relationship did not differ significantly between intact and non-intact families. The school involvement of parents was slightly lower in non-intact families compared to intact families, except for daughters in stepfamilies. We did find that the mother’s life satisfaction was significantly lower in non-intact families, especially in single-mother families.

Consistent with previous research and our expectations, young adults from non-intact families were more likely to have experienced residential mobility and to have a partner, especially young adults from stepfamilies. Furthermore, at the initial observation, men from non-intact families were less often enrolled in general education and more often in vocational education. Employment levels did not differ strongly between family structures at this age.

### Discrete-Time Event History Models

Table 2 (women) and Table 3 (men) present the results of the discrete-time event history models. Model 1 shows that young adults from stepfamilies had about twice the odds of leaving home early than their counterparts from intact families (2.31 for women, 1.97 for men). Those from single-mother families were also more likely to leave early, amounting to a 1.74-fold (women) and 1.61-fold (men) increase in the odds of leaving home early. Model 2 added the variables for the push factors—economic, social, and community resources. For women, we found a curvilinear effect of income on early home leaving. Both women with a household income lower than 100% of the median income and those with an income higher than 150% of the median income were more likely to leave home early than women with a household income of 100–150%. The nonlinear effect of income might be explained by different functions of the household income at different levels of income. At the lower level, income functions as a resource that feathers the nest, whereas it functions as a resource to support independent living at a higher level of income. For men, we only find a positive effect of having a household income higher than 150% of the median income. Living below the poverty line had a significant effect on early home leaving for men when the housing variables were not included; housing explained why men from families below the poverty line were more likely to leave the parental home. In line with Hypothesis 1, poor housing conditions were predictive of early home leaving among women. There was no effect of homeownership. For men, the estimates for the two indicators of housing quality, housing conditions and homeownership, pointed in the expected direction. However, these estimates did not reach conventional levels of statistical significance. A test in which gender was interacted with housing conditions showed that the positive effect of housing conditions on early home leaving was significantly stronger for women than for men.

**Table 2:** Event history models on leaving home early before age 20, women. *N* observations = 4604, *N* individuals = 2250. *Source*: German Socio-Economic Panel 2001–2015, own calculations

	Model 1		Model 2		Model 3		Model 4	
	e^B^	SE	e^B^	SE	e^B^	SE	e^B^	SE
Family structure ref. intact								
Stepfamily	2.31***	0.45	2.12***	0.43	2.12***	0.42	2.03***	0.41
Single-mother	1.74***	0.28	1.31	0.23	1.63**	0.27	1.32	0.23
Age ref. 19								
17	0.26***	0.05	0.25***	0.04	0.27***	0.05	0.26***	0.05
18	0.44***	0.06	0.42***	0.06	0.43***	0.07	0.43***	0.06
Household income ref. 100–150%								
< 60%			1.83**	0.42			1.68*	0.39
60–100%			1.59*	0.30			1.56*	0.30
> 150%			1.64*	0.35			1.78**	0.38
Homeowner			0.94	0.14			0.96	0.15
Poor housing conditions			1.45**	0.21			1.40*	0.20
Quality mother–child relationship			0.89^†^	0.06			0.91	0.07
Parents not involved in school			1.07	0.21			1.12	0.22
Life satisfaction mother			0.92*	0.04			0.93^†^	0.04
Experienced residential mobility			1.17	0.24			1.11	0.23
Main activity ref. employed								
Enrolled in general education					0.26***	0.09	0.28***	0.10
Enrolled in vocational education					0.41*	0.14	0.42*	0.15
Enrolled in higher education					0.18*	0.14	0.21*	0.16
Military/social year					0.24	0.26	0.25	0.27
Not employed, not in education					0.59	0.24	0.57	0.24
Having a partner					1.80***	0.24	1.74***	0.24
Number of children in household	1.27***	0.07	1.24***	0.07	1.27***	0.07	1.24***	0.07
Part of Germany ref. East								
West	0.45***	0.06	0.51***	0.08	0.46***	0.07	0.50***	0.08
Abroad	0.63^†^	0.18	0.69	0.20	0.67	0.07	0.75	0.21
Year of first observation	1.02	0.02	1.03	0.02	1.02	0.02	1.03	0.02
Constant	0.12***	0.02	0.07***	0.02	0.27***	0.10	0.16***	0.07

*e*^*B*^ exponentiated B

^†^*p* < .10; *p < .05; **p < .01; ***p < .001

**Table 3:** Event history models on early home leaving age 22, men. *N* observations = 6669, *N* individuals = 2296. *Source*: German Socio-Economic Panel 2001–2015, own calculations

	Model 1		Model 2		Model 3		Model 4	
	e^B^	SE	e^B^	SE	e^B^	SE	e^B^	SE
Family structure ref. intact								
Stepfamily	1.97***	0.38	1.73**	0.35	2.02***	0.40	1.74**	0.36
Single-mother	1.61**	0.24	1.41*	0.24	1.61**	0.24	1.38^†^	0.24
Age ref. 19								
17	0.31***	0.08	0.31***	0.08	0.25***	0.07	0.26***	0.07
18	0.67*	0.13	0.67*	0.13	0.62*	0.12	0.62*	0.12
20	2.35***	0.41	2.36***	0.42	2.11***	0.38	2.12***	0.39
21	2.91***	0.53	2.97***	0.54	2.45***	0.51	2.53***	0.53
Household income ref. 100–150%								
< 60%			1.34	0.31			1.36	0.33
60–100%			1.21	0.19			1.23	0.20
> 150%			1.92***	0.32			1.83***	0.31
Homeowner			0.80	0.11			0.81	0.11
Poor housing conditions			1.11	0.14			1.12	0.15
Quality mother–child relationship			1.05	0.07			1.04	0.07
Parents not involved in school			1.05	0.21			1.05	0.21
Life satisfaction mother			0.99	0.03			0.99	0.03
Experienced residential mobility			1.78**	0.35			1.87**	0.37
Main activity ref. employed								
Enrolled in general education					0.90	0.22	0.87	0.22
Enrolled in vocational education					0.65^†^	0.15	0.64^†^	0.15
Enrolled in higher education					0.92	0.29	0.84	0.27
Military/social year					1.66^†^	0.48	1.63^†^	0.47
Not employed, not in education					1.18	0.33	1.12	0.32
Having a partner					1.81***	0.22	1.80***	0.22
Number of children in household	1.11^†^	0.07	1.11	0.07	1.10	0.07	1.09	0.07
Part of Germany ref. East								
West	0.66**	0.09	0.64**	0.09	0.65**	0.09	0.64**	0.09
Abroad	0.74	0.22	0.67	0.20	0.74	0.22	0.67	0.20
Year of first observation	1.06**	0.02	1.06**	0.02	1.05**	0.02	1.05**	0.02
Constant	0.04***	0.01	0.04***	0.01	0.05***	0.01	0.04***	0.01

*e*^*B*^ exponentiated B

^†^*p* < .10; *p < .05; **p < .01; ***p < .001

Social resources were measured through three indicators. In contrast to Hypothesis 2, social resources had no effect on early home leaving for men. However, among women, higher levels of mother’s life satisfaction and of the quality of the mother–child relationship were associated with lower odds of leaving home early. A test revealed a significant interaction between gender and the effect of the mother’s life satisfaction.

Lastly, we considered the effect of residential mobility. As expected in Hypothesis 3, young adults who experienced residential mobility were more likely to leave home early. This effect was stronger and significant among men, whereas it was insignificant among women. A test in which gender was interacted with residential mobility showed that the effect of residential mobility was not significantly different for women and men.

In model 3, we looked at the pull factors. In line with Hypothesis 4, young adults who had left the education system were more likely to leave home early than those who were still in education. For women, this applied to all levels of education, whereas for men only to those enrolled in vocational education. Men who joined the military or had a social year were also less likely to leave home early than those who were employed. For both women and men, there was no significant difference in early home leaving between employed young adults and those who were not employed and not enrolled in education. A test showed that the effect of being in the lowest level of education was significantly different for women and men. In support of Hypothesis 5, we found strong pull effects of having a partner both for women and men. Those with a partner were more likely to leave home early, amounting to a 1.80-fold increase in the odds of early home leaving.

Model 4 included all explanatory factors simultaneously. The quality of the mother–child relationship on early home leaving was no longer significant in this model. The other effects in this model did not differ substantially compared to the previous models.

### Mediation Analyses

To evaluate our hypotheses, we tested for mediation effects of the explanatory factors on the relationship between family structure and leaving home using the KHB method. The KHB method provides an unbiased decomposition of direct and indirect effects in logistic regression analyses by rescaling effects. It allows for the comparison of odds by subtracting residuals of the variables that are subsequently added in the original equation. The estimates of these models are more robust than standard logit models. Table 4 (women) and Table 5 (men) give the results of the mediation analyses. We show both the separate indirect effect of each factor based on a model with only that factor and the controls, and the disentangled indirect effect of each factor based on the full model in which all factors were included. All control variables (number of household members, part of Germany, and survey year) were included in all models as concomitant variables.

**Table 4:** Mediation analysis (KHB) of family structure on early home leaving before age 20, women. Percentage explained. *Source*: German Socio-Economic Panel 2001–2015, own calculations

	Stepfamilies		Single mother	
	Only controls	Full model	Only controls	Full model
Economic resources	7.48	3.48	48.96**	27.74**
Household income ref. 100–150%				
< 60%	3.09^†^	1.94	47.97**	30.96**
60–100%	2.84	2.12	10.72**	8.19*
> 150%	− 2.07	− 2.79	− 17.50*	− 24.12**
Homeowner	8.74*	1.37	25.45*	4.11
Poor housing conditions	1.21	0.84	12.02**	8.60*
Social resources	2.54	3.70	17.61**	10.93*
Quality mother–child relationship	2.19	1.25	1.77	1.05
Parents not involved in school	− 0.03	− 0.02	1.61	0.97
Life satisfaction mother	1.53	0.91	14.74**	8.91^†^
Residential mobility	2.63	1.56	2.33	1.41
Main activity ref. employed	2.93	1.82	9.10^†^	7.35^†^
Enrolled in general education	8.39^†^	7.11^†^	12.20^†^	11.21*
Enrolled in vocational education	− 6.12^†^	− 5.53	− 3.30	− 3.80
Enrolled in higher education	− 0.16	− 0.82	2.31	1.61
Military/social year	0.33	0.45	0.71	0.78
Not employed, not in education	0.49	0.61	− 2.82	− 2.45
Having a partner	7.79**	7.18**	2.72	2.56
All mediators		16.19*		49.92**

The effect in the model with only controls is the separate indirect effect when only this variable and the control variables were included, the effect in the full model is the disentangled effect when all variables were included

^†^*p* < .10; *p < .05; **p < .01

**Table 5:** Mediation analysis (KHB) of family structure on early home leaving before age 22, men. Percentage explained. *Source*: German Socio-Economic Panel 2001–2015, own calculations

	Stepfamilies		Single mother	
	Only controls	Full model	Only controls	Full model
Economic resources	13.35*	11.95^†^	23.85^†^	22.09^†^
Household income ref. 100–150%				
< 60%	3.36^†^	2.33	23.80*	15.95
60–100%	2.50	1.99	10.56^†^	8.36
> 150%	− 3.24	− 3.16^†^	− 26.68**	− 25.96**
Homeowner	9.86	9.45	21.85^†^	20.26^†^
Poor housing conditions	1.30	1.34	3.50	3.48
Social resources	− 0.20	0.53	0.62	1.48
Quality mother–child relationship	− 0.25	− 0.18	− 0.36	− 0.25
Parents not involved in school	0.33	0.35	0.53	0.53
Life satisfaction mother	0.36	0.36	1.23	1.20
Residential mobility	8.80**	9.09**	9.61*	9.61*
Main activity ref. employed	− 4.06^†^	− 3.37	2.36	2.92
Enrolled in general education	1.69	1.54	0.29	0.25
Enrolled in vocational education	− 6.12^†^	− 5.64^†^	2.11	1.56
Enrolled in higher education	0.34	0.50	0.37	0.52
Military/social year	− 1.24	− 1.19	− 0.51	− 0.47
Not employed, not in education	0.39	0.42	1.04	1.06
Having a partner	4.69^†^	4.49*	0.98	0.93
All mediators		21.69**		37.32*

The effect in the model with only controls is the separate indirect effect when only this variable and the control variables were included, the effect in the full model is the disentangled effect when all variables were included

^†^*p* < .10; *p < .05; **p < .01

For women, the KHB analyses show that economic resources significantly mediated the effect of earlier home leaving in single-mother families. Taken together, the indicators for economic resources explained 28% of the effect of living in a single-mother family in the full model for women. Both housing quality indicators were significant mediators if tested separately, but only housing conditions formed a significant mediator if both were included. Income had mixed effects; the lowest income levels functioned as mediators, whereas the highest income level functioned as a suppressor. The effect of the lowest income levels was weaker in the full model where also housing was included. Mediation for the effect of living in a stepfamily on early home leaving was weaker and insignificant. The only economic resource that functioned as a significant mediator for women in stepfamilies was homeownership, but this effect disappeared when other indicators were included.

For men, economic resources explained 22% of the effect of living in a single-mother family and 12% of the effect living in a stepfamily. This effect was mainly driven by homeownership, as homeownership explained around 20% of the effect of living in a single-mother family on early home leaving and 9% of the effect of living in a stepfamily. However, for stepfamilies the estimate was not statistically significant. The effect of income resembles the results for women, although here the estimates for the lowest income levels did not reach statistical significance in the full model. These findings support Hypothesis 1a and Hypothesis 1b; economic resources mediate the effect of family structure on early home leaving, especially for single-mother families.

Social resources accounted for around 11% of the effect of living in a single-mother family for women in the full model. This effect was driven by life satisfaction of the mother, which explained 15% of the effect if no other factors were included and 9% in the full model. Again, social resources did not constitute a significant mediator for the effect of living in a stepfamily. For men, social resources were irrelevant as mediators of the effects of both types of non-intact family structures. Overall, we found mixed support for Hypothesis 2.

Residential mobility did not significantly mediate the effect of either type of non-intact family structure on leaving home early for women. However, residential mobility significantly mediated the effect of family structure on leaving home for men, both from stepfamilies (9% explained) and from single-mother families (10% explained). Hence, Hypothesis 3a was supported for men. Yet, we found no support for Hypothesis 3b, in which we expected more explanatory power of residential mobility for young adults from stepfamilies than for those from single-mother families.

The indicators for young adults’ main activity mediated the effect of living in a single-mother family (explaining 7 % of the effect) for women, but not the effect of living in a stepfamily. This effect was driven by the effect of being enrolled in general education instead of being employed. For men, the effect of either type of non-intact family structure on leaving home was not mediated by main activity. This gives little support for Hypothesis 4.

In line with Hypothesis 5a and Hypothesis 5b, having a partner accounted for some of the effect of living in a stepfamily for women (7%) and men (4%). The mediation effect for single-mother families was insignificant.

For the effect of living in a single-mother family, the full model explained a total of 50% for women and 37% for men. For the effect of living in a stepfamily, the full model explained less: a total of 16% for women and 22% for men.

## Discussion

Young adults from non-intact families are more likely to leave home early than those from intact families. Although this finding is well established in the literature on leaving home, few studies have examined what explains this effect. We were able to address this gap in the literature by examining mediation by a range of push and pull factors that are often presumed to differ by family structure and to affect early home leaving. Our analyses explained some of the effect of family structure on early home leaving, but most of the effect remained unexplained. This suggests that the common push and pull factors used in research on leaving home cannot fully explain why young adults from non-intact families are more likely to leave their parental home at a young age.

Economic resources emerged as the most important mediator between family structure and early home leaving. Our findings indicated that young adults from non-intact families were more likely to leave home early because their parental home has fewer economic resources. We examined the effect of parental economic resources not only through parental income, but also through housing conditions and homeownership. Our findings show that housing accounted for a substantial share of the mediation by economic resources. Housing also explained some of the effect of living below the poverty line on early home leaving, young adults from poor backgrounds were (partially) more likely to leave the parental home at an early age because of poor housing. The mediation effects of economic resources were weaker in stepfamilies, which is plausible given that a stepparent often protects against economic deprivation.

In contrast to previous studies (Afifi and Schrodt 2003; McLanahan and Sandefur 1994; Ressler et al. 2017), we did not find significant differences between young adults from non-intact families and those from intact families in the quality of the mother–child relationship and in terms of parental school involvement. Moreover, our analyses showed that these two factors did not affect early home leaving. As a result, social resources did not explain differences in early home leaving between intact and non-intact families. In this regard, the German context of the present study might differ from the US context on which most previous studies are based. There can also be methodological reasons for these differences. We focused on early home leaving and measured relationship quality at age 17, whereas other studies used a wider age range and/or retrospective questions on relationship quality.

Another contribution of our study is that we revealed some notable gender differences not only in the likelihood of early home leaving, but also in the factors that influence early home leaving. Consistent with previous research (Blaauboer and Mulder 2009; Buck and Scott 1993), the effects of economic resources, especially housing conditions, on early home leaving were stronger for women than for men. Moreover, mother’s life satisfaction mattered only for early home leaving of women. An ad hoc explanation for these contrasting patterns could lie in gender differences in time use. Adolescent girls spend more time on housework and caregiving and experience higher supervision by their parents (Wight et al. 2009). Hence, housing conditions and lower life satisfaction of their mother might affect girls more.

We relied on longitudinal prospective panel data which had both advantages and limitations. First, although we could account for the effects of pull factors on early home leaving, we were not able to distinguish between different pathways out of the parental home. Some negative effects of early home leaving might apply only to certain pathways, whereas early home leaving via some pathways might have advantages, such as leaving early for college. We would like to advocate future research to consider the different pathways after home leaving. Robustness checks among a smaller sample of young adults from whom we did know the pathway supported our main findings. Hence, we do not suspect that our conclusions will have to be altered much when distinguishing pathways.

Second, our wide set of measures for social resources was limited to the mother. Previous studies attributed early home leaving among young adults from stepfamilies to higher levels of conflict (Amato and Kane 2011; Cherlin et al. 1995). Our findings suggest that the stepfather–stepchild relationship rather than the mother–child relationship could explain early home leaving among young adults from stepfamilies, as the quality of the mother–child relationship was not poorer in stepfamilies than in intact families. In the absence of a measure for the quality of the relationship to the stepfather, we were unable to examine claims about stepfathers directly. Testing these is an important objective for future research.

Third, our study is one of the first to examine effects of residential mobility in childhood on (early) home leaving. For men, we found that mobility is an important factor in explaining early home leaving and that it mediated the effect of family structure on early home leaving. However, it should be noted that mobility is an indirect measurement for the underlying concept of community resources. Moreover, our measure did not capture multiple moves during childhood, age at leaving home, and distance of moving. Future research should add measures that capture community resources more directly, such as support from neighbors/friends and attachment to the neighborhood.

Our study is one of the first to test explanations for the well-known link between family structure and early home leaving. We combined a youth questionnaire with prospective data of the SOEP from young adults and their mothers. This allowed us to go beyond previous studies and to examine a wide range of explanatory factors. We used KHB analyses to assess not only what factors mediate the effect of family structure on early home leaving, but also the relative weights of the mediators. All in all, our findings pointed to the importance of economic resources as a mediator. However, a substantial part of the effect of family structure remained unexplained, in particular for young adults in stepfamilies. Future research is needed to examine additional factors that influence early home leaving to gain further insight into the complex linkages between family structure and leaving home.
